# An explanation of social conflict? social class and moral foundations. Three meta-analysis

**DOI:** 10.21500/20112084.6456

**Published:** 2025-12-01

**Authors:** David Castilla-Estévez

**Affiliations:** 1 Centro Universitario Cardenal Cisneros. Alcalá de Henares, España. Centro Universitario Cardenal Cisneros Alcalá de Henares España

**Keywords:** Moral foundations, social class, social ladder, social status, group conflict, Fundamentos morales, clases sociales, escala social, estatus social, conflicto intergrupal

## Abstract

Although morality can play an important role in explaining the recent rise of social conflicts inside and outside the Western countries, the study of moral differences between social classes and their possible influence on these social conflicts has been very limited. Thus, this article, which relies on the Moral Foundations Theory, investigates, from a meta-analytic point of view, the relationship of social class (or social ladder, measured as education level, income, and socioeconomic status), and moral foundations, and its possible role as predictor of social conflict. 41 studies (k = 86; N = 285,461), for education level, 26 studies (k = 34; N = 23,889) for income, and 5 studies (k = 16; N = 81,327) for socioeconomic status, were selected. Results showed how all pooled correlations are either statistically not different from zero or of negligible size. Limitations regarding a restrictive range of available data, are discussed.

## Resumen.

Si bien la moral puede desempeñar un papel importante a la hora de explicar el reciente aumento de los conflictos sociales dentro y fuera de los países occidentales, el estudio de la relación entre moral y clase social como predictor del conflicto ha sido limitado. Este artículo se basa en la Teoría de los Fundamentos Morales para investigar, desde un punto de vista meta-analítico, la relación entre clase social (o escala social, medida como nivel educativo, ingresos y estatus socioeconómico) y fundamentos morales. Se seleccionaron 41 estudios (k = 86; N = 285.461), para nivel educativo, 26 estudios (k = 34; N = 23.889) para ingresos y 5 estudios (k = 16; N = 81.327) para nivel socioeconómico. Los resultados mostraron correlaciones de tamaño insignificante para todos los fundamentos morales. Se discuten las limitaciones relacionadas con la restricción de rango de los datos disponibles.

## 1. Introduction

Can morality explain, at least in part, the current rise of inter-class social conflict? Do people from different social status exhibit different moral matrices? Together with the absence of institutional leaders- hip, economic inequalities are one of the main factors that can facilitate the emergence of social disorder (Reicher & Stott, 2020), and these two elements have been strongly presented in recent years in Wes tern societies, especially since the economic crisis of 2008 (Evans, 2017). During this time, there has been an increase in polarization in the structure of social classes, due to an increase in economic in- equality and a shrinking middle class in Western countries (Chauvel et al., 2021; Scott & Pressman, 2011). As a result, there is growing frustration and resentment towards institutions and a backlash against elites from certain sectors of the population, especially from the working class (Jedwab et al., 2020; Jetten et al., 2020; Keiger, 2020; Saunders, 2022), that have led in part to the rise of support for populist and hard right parties inside and outside the Western countries (Engler & Weisstanner, 2021; Grigoriadis, 2020; Masood & Nisar, 2020; Weisskircher, 2020).

The awareness that elites and upper classes do not share the same interests, nor do they share their values or vision of society as the people, plays a prominent role in this growing resentment towards institutions and elites (Grigoriadis, 2020; Masood & Nisar, 2020; Taguieff, 2016). These differences of interests and values between social classes can be also found measurable and objective, since the differences in how elites and masses conceive democracy, meritocracy, diversity and li beral norms in general, have been widely reported by scholars (e.g. Aspinall et al., 2020; Atria et al., 2020).

Although various articles have already studied the relationship between social class and the emergence of social disorder (e.g. Evans & Opacic, 2022; Keefer et al., 2015; Owens et al., 2010), and also recent studies have investigated the relationship between social class and moral principles (e.g. De Keere, 2020), the study of moral differences between classes and their possible influence on these social conflicts has been very limited to date.

As a result, and drawing on Moral Foun- dations Theory (MFT), this article investigates, from a meta-analytic point of view, the relationship of social class (in a broad sense, as it is about to be explained), and moral foundations. The MFT relates higher levels of moral relevance or moral judgement with stronger emotional reactions; which could lead to higher levels of conflict intractability among groups with different moralities. This theory was originally meant to explain how different cultures exhibit different moralities (Haidt & Joseph, 2004) and also explore moral differences between liberal and conservative ideologies and the emergence of social conflict it could facilitate (Graham et al., 2009). Therefore, if there are different moralities for different people depending on their social class, social conflicts between classes could be, at least partly, explained by the MFT.

### 1.1 Social Class as Social Ladder

To study the relationship between morality and social class properly, “social class” must be adequately defined. In this sense, a suitable definition could be which was proposed by Kraus et al. (2012), for whom rather than as a characteristic of the in dividual, social class, from a rank-based perspective, is a context the individual in- habits and to which they must adapt.

For this reason, it is necessary to include not only objective measurements of material resources, such as income and education, but also subjective measures of social status in relation to others when measuring social class (Kraus et al., 2012; Liu et al., 2004). Subjective social sta tus can help to capture relevant psycho- logical variables, like influence in social groups and mental well-being (Anderson et al., 2012) and it should be included along with income or education level, for these three variables do not always co rrelate with each other (Anderson, 2018).

Thus, to avoid confounding this concept with the classic Marxist concept of “social class”, which is more restricted (Engels & Marx, 1848/2019), a broader, and probably more suitable one for so cial psychology, “social ladder”, used by Kraus et al. (2013), is used onwards. Finally, three social ladder-related varia bles, education level, income level and subjective socio-economic status (SES), are considered.

### 1.2 Moral Foundations Theory

As Graham et al. (2013) explain in the article “Moral Foundations Theory: The Pragmatic Validity of Moral Pluralism”, the MFT states that human morality comprises a set of moral intuitions that precede mo ral reasoning and are primal to moral judgement. This happens in such a manner that moral reasoning is only meant to confirm in most of the cases the intuitive judgement already done automatically by the individual. Moral intuitions have been evolved through generation as a group adaptation to the environment. Each group has developed over time a specific moral configuration or moral matrix that may be more or less different from other groups' depending on the evolutionary and geography context. Consequently, each person is born with a first draft of his/her own cultural or geographical group's moral matrix. Moral intuitions are classified on five groups or Moral Foundations: Harm/ care (HC), Fairness/cheating (FC), Loyalty/ betrayal (LB), Authority/subversion (AS), Purity/degradation (PD). Whereas the first two foundations (HC and FC, also known collectively as Individualizing Foundations or IF), refer to interpersonal relations and can be found more or less in every human society, LB, AS and PD (also known collectively as Binding Foundations or BF), refer to specific group social and cultural norms that are seen as moral by group members. Thereby, Loyalty/ betrayal is specifically about loyalty to one's group over other peoples and groups, whereas Authority/ subversion refers to the submission to one's group's hierarchical order and to act accordingly to the role each one's have in it. Finally, Purity/degradation refers to the assumption that some things, opinions or behaviors (regarding sex, food, the human body, national symbols, the environment, etc.) are considered as sacred by the group, and therefore must not be at- tacked or subverted under the group's moral norms. Another individualizing foundation, Liberty/oppression (LO), was included afterwards (Iyer et al., 2012). However, it has been scarcely investigated to date.

One of the most important consequences the plurality of moral matrices across different groups have according to the MFT, is that group conflict is significantly likely between two groups that do not share the same moral matrices. For example, evidence was found confirming that the social confrontations derived from politics corres ponded to a moral gap between liberals and conservatives (Graham et al., 2009), which has been found in many countries like Fin- land (Kivikangas et al., 2017), Turkey (Yilmaz et al., 2016) or New Zealand (Davies et al., 2014). The study was submitted to and approved by the ethics committee of the San Ig nacio University Hospital (FM-CIE-044-2021) and informed consent was obtained from all individual participants included in the study.

This plurality of moral matrices has also been found at a regional level. It is already known that nations that have encountered more ecological and historical threats, namely Eastern countries, tend to show stricter social (group) norms than Western countries (Gelfand et al., 2011). This result has been verified from the MFT too. For example, Graham et al. (2011), using a large sample of 107,151 people, found that Western participants showed significantly lower levels of Loyalty and Purity than Eastern countries.

As it seems, higher group struggle might have facilitated higher levels of (at least) some moral foundations within the group's moral matrix. Could this mean that lower status people could also have developed mo ral matrices with higher moral foundations levels compared to higher status people?

### 1.3 Social Ladder and Morality

The relationship between education level, income, and socioeconomic status has been shown in various studies (Belsky et al., 2018; Kim et al., 2015; Wright & Perrone, 1977), and their relationship with human morality has also been investigated, although the number of studies involved had been scarce, and results have been contradictory in part. For example, while some studies have defended that people from upper class show more egoism (Coté et al., 2013; Dubois et al., 2015) and that lower-class people are more generous (Piff et al., 2010), other studies say that children from rich countries are the most generous (Cowell et al., 2017) and that lower-class people develop more prejudices (Carvacho et al., 2013). Conversely, the relationship between socio-demographic status and moral development has been found in some studies (Commons et al., 2006; Koenig et al., 2004) but not in others (Vera-Estay et al., 2016), and same for the relationship between education and moral reasoning, which has also been found in some studies but not in others (Cesur & Topgu, 2010; Doyle & O'Flaherty, 2013). Differences in moral values between different social ladder steps were already found by Haidt et al., (1993) between low SES individuals and high SES individuals both in Brazil and the US. In addition, there are some studies that expose that people in the lower steps, since they live in a situation of greater insecurity and with less access to resources, tend to develop more community values and self-concept. On the contrary, people in higher social ladder positions, develop values and self-concept which are more related to individual autonomy (Everett, 2003; Kohn & Schooler, 1969; Coté, 2011; Kraus et al., 2012; Piff et al., 2016). However, other studies have not found such a relationship (Miles, 2014; van Leeuwen et al., 2014), and when studying specifically which values people from the lower and upper sections of the ladder differ in, the results are neither conclusive for the principle of authority (Coté, 2011; Kohn & Schooler, 1969; Kraus et al., 2012) nor for harm avoidance (Horberg et al., 2009; Kraus et al., 2012). Purity has produced more coherent results, being a more relevant moral issue for lower positioned people than for upper positions ones (Haidt et al., 1993; Horberg et al., 2009; Kraus et al., 2012; van Leeuwen et al., 2014). Given all the gathered evidence, the question if there are moral differences along the social ladder, and which they are, is far from being answered.

### 1.4 Framework Justification and Hypothesis

Despite the amount of research done about possible moral differences between people from different steps of the social ladder, results have not been conclusive until now. Therefore, the present article aims to gather all the available data produced within the framework of the MFT and investigate it from a meta-analytical approach, since the amount of data already produced in this regard can lead to a higher confidence in a metanalysis-based result. The MFT is probably the most convenient moral psycho- logy theory to choose for a metanalytical approach, since has been utilized or at least cited in more than 6000 articles in the last decade, and it has produced at least three meta-analysis to date, regarding age (Castilla-Estévez & Blázquez-Rincón, 2021), ideology (Kivikangas et al., 2021) and religiosity (Saroglou & Craninx, 2021). Moreover, the reliability of its main questionnaire, the the MFQ30, has been proven extensively with massive worldwide-based samples (Graham et al., 2011).

The research hypothesis of this study is the following: given that higher group struggle might have facilitated higher levels of (at least) some moral foundations within the group's moral matrix, we expect lower-class people to exhibit significative higher concerns for binding foundations (LB, AS and PD) than higher-class individuals.

## 2. Method

The present work studies, from a meta-analytical perspective, the relationship between social ladder and moral foundations. This study followed mainly APA's Preferred Reporting Items for Systematic Reviews and Meta-Analyses (PRISMA) (Page et al., 2021) guidelines.

### 2.1 Search Strategy and Study Selection Criteria

Since the number of possible combinations between social ladder (three variables) and moral foundations (eight variables) is quite high, and therefore the number of hypothetical explanations of a moral foundations-based social conflict are also quite high, it was necessary not to miss any piece of data available in the selection of data process. For example, greater levels of every moral foundation associated with lower levels of inco me, but not to lower levels of SES, would mean that social conflict between social classes is mainly associated with economic struggle, and maybe not so much to the social class itself. This result would yield different policy implications, for example, moral differences between different levels of income are also found for SES and for the education level. Therefore, the aim of the selection process was to collect as much data as possible from studies including measures from any of the six moral foundations (or any of the two moral groups, IF and BF) and also measures of the three social ladder related variables (education level, personal income level and/ or a measure of subjective SES). However, it was necessary to exclude data produced after an experimental manipulation, because then the relationship between social ladder and moral foundations could depend to the manipulation itself.

Thus four exclusion criteria were followed: (1) moral measures taken under manipulated circumstances or experimental treatments were excluded; (2) samples comprised psychopathically diagnosed individuals were excluded; (3) to ensure sampling independence, when one sample was utilized in more than one article, only the first article to use that sample was chosen, and the rest were excluded; (4) articles which utilized a MFT-based moral model not comprised, as proposed originally by Graham et al. (2009), by five or six separated moral foundations (Harm/care or HC, Fairness/cheating or FC, Liberty/oppression or LO, Loyalty/ betrayal or LB, Authority/subversion or AS, and Purity/degradation or PD) or two moral groups (Individualizing Foundations or IF, com- posed by HC, FC and LO, and Binding Foun- dations or BF, composed by LB, AS and PD).

Pearsons's correlations between each of the moral foundations (or moral groups) and each of the three social ladder variables were utilized as the effect sizes (ES) for the metanalyses. Although it can be stated that Education Level, Income and SES are often measured as ordinal variables, and therefore it would be more accurate to utilize non-parametric correlations as the primary effect size for the study, there are at least two reasons why Pearson's correlations were chosen as effect sizes over Spearman's correlations: (1) the vast majority of articles with data available regarding the relationship between moral foundations and education level, income or SES utilized Pearson's correlations, and not Spearman's; (2) both income and SES can be seen as continuous variables, even though they can be usually presented in an ordinal fashion. There- fore, a linear association between moral foundations and income or SES can be expected, and it can be preferable to utilize the same effect size also for Education Level, in order to facilitate interpretation of the results.

The search of studies up to 2018 was done in PubMed, Web of Science, Psychinfo and Scopus with the terms “moral foundations” and “Haidt”. The search was also made in Google Scholar, in order to find other possible non-published works. The selection process was initially made by two researchers. However, sin ce one researcher left at the very beginning of the project, the whole process had to be made almost entirely by me. In order to minimize the possible risk of bias in the process of study selection, the process itself was made independently twice, in the first trimester of 2019 and the third trimester of 2019, and the few discrepancies found were again revised and corrected. A complete revision of the whole process was made at the beginning of 2020 to ensure that selection criteria had been applied correctly throughout the whole selection process. Lastly, a final search was again made in June 2024 in PubMed, Web of Science and Scopus to ensure that no article of interest had been missed with the previous searches.

**Figure 1.Flowchart:**
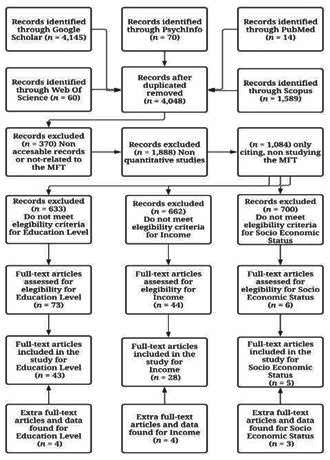
of the studies' search.

A total of 73 articles for education level, 44 articles for income, and 6 for SES were first selected. Pearson correlations with each moral foundation or group was presented on 19 articles for education level, 15 articles for income, and 2 for SES. For all the rest of the articles, mails to the authors were sent, and data were received from 20, 9, and 0 articles for education level, income, and SES, respectively. Additional available data from other 4, 4, and 3 articles for education level, income, and SES, respectively, received from their authors in previous meta-analytical projects, were included afterwards. Our final database for education level was comprised 43 studies (k = 107; N = 297,744), for income, it was comprised by 28 studies (k = 65; N = 32,040), and for SES, it was comprised by 5 studies (k = 16; N = 81,327). The complete selection process was recorded in an excel file and a summary was saved in a word document afterwards. More detail about this process is shown on Figure 1.

### 2.2 Coding of the Studies

Pearson correlations was coded positively. Positive correlations mean that higher-social ladder people (higher education level, higher income, and higher socioeconomic status) present higher moral foundations' levels, and vice versa. Since a large heterogeneity for pooled correlations was expected, some possible moderating variables were included in the analysis. First, gender (coded quantitatively as the percentage of females in the sample) was included, as gender differences in moral foundations has been found before (Atari et al., 2020). Second, political ideology (coded quantitatively from 0 = extreme liberal to 1 = ex treme conservative) was included since it has been found to be significantly related to moral foundations (Graham et al., 2009). Third, possible culture differences were analyzed by co- ding the US variable as a possible moderator, separating US-based samples and non-US-based samples, because US peoples' traits have been found to be the least generalizable com pared to other countries (Henrich et al., 2010). Several studies, from which the whole data- set was available, included participants both from the US and outside the US (Cantarero et al., 2018; Graham et al., 2009; Graham et al., 2011; Klein et al., 2015). In those cases, the dataset was split into samples, regarding the country (or world region) or origin. Samples with less than 30 participants, were excluded. Fourth, since moral foundations are measured with several different instruments, an MFQ30 variable was created in order to sort out any possible methodological bias in our interpretation of the results. Samples using the most utilized moral foundations questionnaire, the 30- item version (MFQ30) (Graham et al., 2011) was coded as “1”, and the rest of samples were coded as “0”. Fifth, the YM variable, which separates samples gathered by the MFT authors and collaborators in the YourMorals.com site from the other samples, were both finally included to sort out any sampling bias in the results. Finally, mean age of the sample was also included. Descriptive information is not presented here, but it can be found as supplementary information in https://osf.io/95zne/

### 2.3 Statistical Analyses

Before the analyses themselves, possible outliers were searched, and their effect was con- trolled, using the box-plot analysis for univariate data (Mosteller & Hoaglin, 1991) in order to ensure validity to our results interpretations (Viechtbauer & Cheung, 2010).

Fisher's Z-transformation was calculated for each Pearson's correlation in order to normalize its distribution and stabilize its variances. Once pooled, Fisher's Z and confidence intervals were calculated, Fisher's Z values were transformed back into the Pearson's correlation metric to facilitate the interpretation of the results (Borenstein et al., 2009) for each of the moral foundations HC, FC, LO, LB, AS, and PD or the two moral groups, IF and BF. Random-effects model was taken, as a large heterogeneity among pooled correlations was expected (Borenstein et al., 2009; Sánchez-Meca & Marín-Martínez, 2008), and each pooled correlation heterogeneity was tested with the Q and *I 2* statistics (Huedo-Medina et al., 2006) for every moral foundation or moral group. Publication bias analyses comprised by a Kendall's Tau adjusted rank correlation test (Begg & Mazumdar 1994) and Egger regression analyses (Egger et al., 1997) were afterwards computed for every moral foundation and group. When necessary, publication bias was corrected with the Trim & Fill procedure (Duval & Tweedie, 2000). Third, for meta-analyses with at least 30 estimates, moderator analyses were conducted through simple meta-regression analyses for continuous variables. Since US, MFQ30 and YM are dichotomic, they were included also in the regression analyses as dummy variables to facilitate interpretation and to carry out multiple regression analysis-based explanatory models for each moral foundation afterwards. Coefficient R2 for meta-regressions was estimated to address the percentage of specific variance explained by each model (Borenstein et al., 2009). Fourth, explanatory models with significant predictors, significant associated Q statistics, and a non-zero R2 coefficients, were finally selected. Analyses were per- formed using Wilson's meta-analysis macros for SPSS (Wilson, 2005) and metafor package for R (Viechtbauer, 2010) .

## 3. Results

### 3.1 Pooled Estimates and Heterogeneity

Pooled ES analysis for education level, in come, and SES, including the number of in- dependent estimates, the sample size, the pooled correlations, and its 95% confidence interval, the t2 estimate, the Q-test, and the the *I2* statistics, both for the whole world and for the US, can be found in Table 1.

**Table 1:** General results por every foundation metanalysis.

*MF*	K	N	r+	95%Cl	T^2^	^Q^	*I* ^*2*^
*Economic Level*							
HC	71	287,216	0132	[.0008; .0255]	.00112	480.01***	85.4
FC	70	285,530	.0247	[.0118; .0377]	.00120	250.83***	72.5
LB	70	285,448	-.0798	[-.0978; -.0617]	.00347	593.53***	88.4
AS	70	285,530	-.0898	[-.1083; -.0712]	.00379	673.88***	.89.8
PD	71	287,052	-.0779	[-.0945; -.0613]	.00284	507.05***	86.2
LO	2	661	-.1469	[-.2974; .0108]	.00918	3.104***	67.8
IF	33	8,944	-.0079	[-.0351; .0194]	.00129	42.284	26.7
BF	32	7,743	-.0377	[-.0794; -.0042]	.00760	82.837***	61.4
*Income*							
HC	31	21,365	-.0394	[-.0580; -.0207]	.00096	49.273*	39.1
FC	30	20,445	-.0663	[-.0911; -.0415]	.00262	78.831***	63.2
LB	30	19,984	.0242	[-.0006; .0490]	.00261	77.865***	62.8
AS	30	20,445	.0321	[.0070; .0572]	.00274	81.465**	64.4
PD	31	21,332	-.0046	[-.0289; .0197]	.00264	82.832***	63.8
LO	2	1,154	-.0076	[-.0965; .0814]	.00322	4.229	52.7
IF	35	10,560	-.0503	[-.0694; -.0312]	.00000	32.237	.0
BF	36	10,722	.0474	[-.0147; .1092]	.02873	312.70***	88.5
*Socioeconomic Status*							
HC	16	81,327	-.0358	[-.0644; -.0072]	.00223	97.349***	84.6
FC	16	81,327	-.0493	[-.0761; -.0223]	.00188	84.417***	82.2
LB	16	81,327	.0547	[.0202; .0891]	.00366	151.08***	90.1
AS	16	81,327	.0386	[.0123; .0649]	.00176	79.858***	81.2
PD	16	81,327	.0101	[-.0223; .0425]	.00310	129.33***	88.4

*Note*. MF = Moral Foundation; HC = Harm/ care; FC = Fairness/cheating; LB = Loyalty/ betrayal; AS = Authority/subversion; PD = Purity/degradation; k = number of independent samples; N = total sample size; r_+= pooled correlation estimate; 95% CI = confidence interval; t2 = between-study variance; Q = Cochran's heterogeneity Q statistic with k-1 degrees of freedom; I2 = heterogeneity percentage index. * p < .05, ** p < .01, *** p < .001. Table 1 shows how both for education level, for income, and also for socioeconomic sta tus, all pooled correlations are either statistically not different from zero or of negligible size, always below 0.1. These results stand across the three variables of study: education level, income, and socioeconomic status. On the other hand, heterogeneity is, in most of the cases significant, and reaches values between 80-90% for education-level-based and socioeconomic-status-based correlations. Heterogeneity is the lowest for income-based correlations, ranging in all cases (except for BF) below 70%, IF correlations do not show a significant heterogeneity, for either education level nor for income.

**Table 2:** Publication bias analyses: Kendall's Tau and Eg- ger regression statistics for moral foundations correlation with Education level, Income and SES

	Eduaction Level		Income			SES
	Kendall's		Kendall's		Kendall's	
MF	Tau	Regression	Tau	Regression	Tau	Regression
HC	-.1328	-3.347***	-.0302	-.1170	.0500	-.0022
FC	-.0725	-3.588***	-.1151	-.6647	.1000	-.0105
LB	-.1197	-2.203*	.0276	.5403	-.1000	2.269*
AS	-.1943*	-2.415**	-.1381	-2.078*	.1833	1.661
PD	-.1866*	-2.661**	-.0460	-1.393	.0833	1.639
IF	.0777	.5731	.0960	1.718		
BF	.0222	2.243*	.1812	-.6002		

*Note*. MF = Moral Foundation or group; HC = Harm/care; FC = Fairness/cheating; LB = Loyalty/betrayal; AS = Authority/subversion; PD = Purity/degradation; LO = Liberty/ oppression; IF = Individualizing Foundations group; BF = Binding Foundations group; * p < .05, ** p < .01, *** p < .001; + = it was not possible to run the analysis due to lack of data.

### 3.2 Analysis of Publication Bias

Both Kendall's Tau adjusted rank correlation test and Egger regression analyses were carried out for each of the five foundation and for each of our three social ladder va riables: education level, income, and socioeconomic status, in order to test possible validity threat publication bias might produce on Table 1 results. As Table 2 shows, there are some significant results, which indicate a possible publication bias in the results shown on Table 1. While the significant results found in LB (SES), AS (Income) and BF (Education Level) for the Egger regression test, are quite weak and are not detected by the rank correlation test, the biases found in Education Level for HC, FC, AS and PD are quite clear and pose a risk to our results. Thus, trim and fill correction analyses were carried out. Results yielded similar pooled correlations for HC *(r*_*+*_ = .0346; 95%CI = [.0225; .0467]), for FC (*r*_+_ = .0539; 95%CI = [.0402; .0676]), for AS (*r*_+_ = -.0900; 95%CI = [-.1087; -.0713]), and for PD (*r*_+_ = -.0575; 95%CI = [-.0753; -.0397]).

### 3.3 Moderator Analyses

Apart from IF, every other pooled correlation showed a significant amount of heterogeneity on Table 1. In order to identify at what extent our moderator variable candidates could explain this heterogeneity, simple meta-regressions, taking correlations between each moral foundation and education level, and each moral foundation and income, as the dependent variables, were carried for meta-analyses with at least 30 estimates. As a consequence, analyses for SES were not computed. Categorical variables were treated as dummy variables in order to compare more easily the different moderating effects of categorical and quantitative variables, and also in order to build explanatory models for each moral foundation pooled correlation. For significant moderators, a series of explanatory multi-regression models were computed. Only explanatory models with significative predictors, significant associated Q statistics, and a non-zero R^2^ coefficients, were finally selected.

Since the number of analysis of moderators are quite large, and most of them yielded non-significant results, only the explanatory models selected for Education Level and Income are presented here. The complete results for moderator analyses can be found as supplementary material in https://osf.io/95zne/

### 3.4 Explanatory Models

Once every moderator candidate has been tested, every significant explanatory model for each correlation regarding social ladder and moral foundations is presented in detail. Note that no model is presented when no significant moderating variable that also explain some amount of variance has been found.

**Table 3:** elected explanatory models for correlations be- tween each moral foundation and each social lad- der variable (Education Level and Income)

*MF*		*B 95%Cl*		*Z*	*β*	*I* ^*2*^	*R* ^*2*^
Education Level							
HC	Constant	-.023	[-.038, -.009]	-3.20**	.000	49.4	63.4
	YM	.78	[.059, .097]	7.99***	.663		
FC	Constant	-.008	[-.025, .010]	-.856	.000	64.2	27.5
	YM	.064	[.041, .088]	5.31***	.471		
AS	Constant	-.089	[-.128, -.050]	-4.50***	000	87.8	8.4
	YM	.081	[.047, .121]	4.49***	.350		
	MFQ30	-.052	[-.093, -.012]	-2.54* -	.198		
PD	Constant	-.017	[-.074, .040]	-.581	.000	86.4	11.0
	Ideology	-.171	[-.323, -.020]	-2.21* -	.234		
BF	Constant	-.279	[-.065, -.009]	-1.48	.000	59.3	56.4
	US	-.095	[-.185, -.006]	-2.09* -	.368		
Income							
FC	Constant	-.021	[-.058, .017]	-1.07	.000	50.9	38.6
	US	-.069	[-.114, -.023]	-2.94**	-.515		
LB	Constant	.038	[.015, .060]	3.28**	.000	47.0	45.0
	YM	-.110	[-.172, -.048]	-3.45***	-.537		
AS	Constant	-.007	[-.047, .034]	-.3169	.000	56.9	25.8
	US	.059	[.010, .108]	2.38*	-.367		
PD	Constant	-.034	[-.071, .003]	-1.819	.000	65.9	3.8
	MFQ30	.050	[.002, .098]	2.059	.319		

*Note*. MF = Moral Foundation; HC = Harm/ care; FC = Fairness/cheating; LB = Loyal- ty/betrayal; AS = Authority/subversion; PD = Purity/degradation; BF = Binding Foundations; Gender = percentage of wo- men; YM = sample from YourMorals.com; N = Sample size; US = the sample comes from the US; B = regression coefficient; 95%CI = 95 %confidence interval for B; Z = test for B significance 3 = standardized regression coefficient; I2 = heterogeneity index for the model; R2 = Variance explained by the model. * p < .05, ** p < .01, *** p < .001

As it is shown on Table 3, after controlling the effect of the rest of variables, YM, US, MFQ30 and Ideology still have a significant role in explaining the heterogeneity of the pooled ES. YM and US, are the most important moderators, though, since they are present in a larger number of explanatory models as predictors, and also, they explain a higher amount of pooled ES heterogeneity.

With regard to Education Level, YM has a positive effect on the pooled ES for HC, FC or AS, whereas US has a negative effect on the pooled ES for BF. Specifically, for people coming from YM samples, higher levels of education are related to higher HC, FC, AS levels. On the contrary, for people recruited outside the Your Morals platform. higher levels of education are related to lower HC, FC, AS levels. Lastly, higher levels of education are more strongly related to lower BF levels in US samples, compared to non-US samples.

With regard to income, YM has a significant and negative effect on the correlation between LB and income, while US has a significant and negative effect on the correlation between income and HC, and a significant and positive effect of the correlation between AS and income. These results mean the following: first, a higher in come is related with lower concerns for LB in YM samples, whereas a higher income will be related with higher concerns for LB in non-YM samples. Second, wealthier people will show lower levels of HC than poorer people in the US, but this effect would not be found outside the US. Finally, the effect of US on the correlation be- tween AS and income is positive. This means that US wealthier people will show higher levels of AS than US poorer people, but this effect would not be found outside the US.

## 4. Discussion

The results obtained show that moral foundations correlate in a quite negligible or non-significant way with social ladder. This outcome stands regardless the way social ladder is measured: by educational level, income, or SES. As a consequence, if there is no moral divi de between social ladder position, then class conflict may not be itself a mo ral issue, at least from the MFT perspective. However, some correlations are still significant, though very small, and there are some differences between results for education level and results for income. Lastly, analysis of moderators points out to a probable effect of cultu re on those differences.

### 4.1 There is no evidence for a social-ladder-based moral matrix

There is no main effect of social ladder on people's moral matrix. Results show that neither educational level, the income level, nor the SES correlate in a relevant way with any of the moral foundations or moral group. Overall, despite the fact that the pooled correlations appear significant on some occasions, with values ranging between -0.900 and 0.547, in none of them the correlation reaches 0.1 in absolute value. This means that social ladder in no case explain more than 1% of the variability of people's moral levels, which, taking also into account that the large sample sizes endowed the analyses with great statistical power, allows to conclude that the correlations found between social ladder and moral foundations may be just a product of statistical power, rather than a relevant effect.

Moreover, heterogeneity levels are much smaller for income than for education level or SES. Given the great differences in sample size among these three variables, specially between education level and income, and the fact that almost none of our candidate variables has been found to have a moderating effect on the pooled correlations (with some exceptions that are discussed be- low), it can be stated the following: contrary to what some found in the past, including Haidt himself (Haidt et al., 1993) social ladder does not play a relevant role in explaining moral differences between genders, age groups, conservatives and liberals, and region overall. However, the small size of the significant pooled correlations could indicate that, although in general terms there is not a relevant effect to consider, this effect might appear under very specific circumstances. This is what the analyses for moderators might show, as they yielded some results that are worth commenting briefly.

### 4.2 Culture may be the most important moderator

These two variables can be connected to culture differences in moral foundations, although the effect YM had on the pooled ES can also indicate a specific sampling bias of the Your Morals platform.

The effect of culture predicted by Haidt (2001) on how moral foundations develop over time, and the effect US weirdness has compared to other countries (Henrich et al., 2010) appear here regarding moral differences by education level and also by income. Furthermore, since YM samples also show some westernized characteristics, like the use of on-line platform to fill out a questionnaire in English, independently of the native language of the participant, the effect YM had on the pooled ES could suggest possible cultu ral heterogeneities within each region due to westernization (or, again, weirdness).

In this regard, the results obtained show that the differences in moral foundations between people with higher and lower education levels, and also between poorer and richer people, could depend significantly on the socio-cultural context of the subject. However, the way the context influences the relationship between social class and moral foundations is not clear. For example, YM appears to have a negative effect on the pooled correlation between LB and Income, but it has a positive effect on the pooled correlation between AS and Education Level. Also, while US has a negative effect on the pooled correlation between AS and Income, it has a positive effect on the pooled correlation between PD and Binding Foundations. Taken together, the effects YM and US have on the pooled ES are quite difficult to interpret, since they do not maintain the same direction for YM and US in every case, and neither they maintain the same direction for Education Level and Income. Moreover, although the models involving both US and YM explain a high amount of heterogeneity of the correlations, ranging from 8.4% to 63.4%, neither both moderators make any correlation reach clearly above the 0.1 mark. Thus, and despite the effect both US and YM can have on the pooled ES, the relationship between social class and moral foundations is still quite weak.

Finally, the role of YM as a moderating va riable it is also a matter of concern. Since some results depend on the platform for recruiting participants, there is a potential validity risk for their generalizability when conducting MFT studies based solely on data from the YourMorals.com platform. This bias has also been detected and discussed in a previous meta-analysis regarding the relationship between ideology and moral foundations (Kivikangas et al., 2021).

### 4.3 Limitations and Future Directions

The adequate prediction this article makes regarding the relationship between social lad- der and morality is one of its greatest strengths. Probably counterintuitive to many other works (vid. Supra), social ladder and the moral matrix are not directly related. These results are quite generalizable, moreover, because they were obtained from databases made up of samples from the five continents and ranging from a thousand to more than two hundred thousand people.

However, the article suffers from several limitations that future studies will have to address. First, more data and studies regarding SES and people outside US are necessary. There are not enough published data available that could allow to investigate the effect moderating variables could have on SES-moral foundations correlations. Moreover, the vast majority of data came from the US. Therefore, it was not possible to investigate differences between different regions.

Second, our results suffer from a restrictive range regarding both education level and in come. Most of available data came from lower medium-class to upper-medium class people. This means that the results presented here may not be replicated in samples with a high percentage of non-educated people or really wealthy people, for example. This could explain why results for PD do not reflect Haidt's results (Haidt et al., 1993). Therefore, more research focused on the relationship between moral foundations and social ladder is needed to have a real clear picture of the relationship between these two constructs.

Third, it is also possible that our results regarding the moderating role of US and YM, could reflect a possible bias in the way the MFQ30 items are written, which in turn could mean that some moral foundations could have different moral meaning for people for different cultures and populations. Although Graham et al., (2011) showed a quite strong fit for the MFQ30 for samples form all around the world, other authors, not related to the YM platform, have found some convergence problems for the five-factor model of the MFT in black samples (Da- vies et al., 2016), religious samples (Davies et al., 2017), and samples from non-western countries (Iurino & Saucier, 2020). Furthermore, the use of a moral instrument other than the MFQ30 can also lead to some biases, as it was shown in Table 3.

Lastly, the risk of selection or codification bias due to the fact that the whole selection and codification processes have been carried out by just one researcher cannot be entirely ruled out, although the general results seem to be quite clear and non-depending on a specific selection of samples, as the small changes on the pooled ES after the correction yielded by the trim and fill procedure might suggest.

## 5. Conclusions

Although it may be counterintuitive for some, social ladder does not influence our moral matrix directly. This may contradict some of the results obtained in this regard by various researchers, but at the same time it can serve as a global result that overcomes the contradictions found in the results of said studies. The present study does not answer many questions, but it does provide a basis from which to study how the social context influences the morality of the individual, and that is probably its greatest virtue.

However, and given the potential risk of bias regarding the MFQ30, future similar investigations within other theoretical frameworks would be necessary in order to confirm or refute if conflict between social classes is related (or not) with human morality. Two interesting alternative theories would be suggested. First, the neo-Kohlbergian model, which focuses more on moral reasoning than on moral intuitions (Rest et al., 2000), could show different stages of moral reasoning between people with high education level and low education level. Second, the CNI (consequences, norms, and generalized preference for inaction versus action in moral dilemmas) model of moral decision-making (Gawronski et al., 2017), found that women show a stronger sensitivity to norms and a stronger general preference for inaction than men. This model could hypothetically investigate if this gender difference could explain the moderating effect gender had on the relationship between education and HC.
